# Antigen-specific single B cell sorting and expression-cloning from immunoglobulin humanized rats: a rapid and versatile method for the generation of high affinity and discriminative human monoclonal antibodies

**DOI:** 10.1186/s12896-016-0322-5

**Published:** 2017-01-09

**Authors:** Laure-Hélène Ouisse, Laetitia Gautreau-Rolland, Marie-Claire Devilder, Michael Osborn, Melinda Moyon, Jonathan Visentin, Frank Halary, Marianne Bruggemann, Roland Buelow, Ignacio Anegon, Xavier Saulquin

**Affiliations:** 1 INSERM Center for Research in Transplantation and Immunology (CRTI) U1064; Université de Nantes; Centre Hospitalier Universitaire de Nantes Institut de Transplantation Urologie Néphrologie (ITUN), Nantes, F44000 France; 2 Transgenesis Rat ImmunoPhenomic Platform Structure Fédérative de Recherche François Bonamy Centre National de Recherche Scientifique UMS3556, Nantes, F44093 France; 3 CRCNA UMR S892 INSERM 6299 CNRS Université de Nantes; Université de Nantes Faculté des Sciences et Techniques, Nantes, F44093 France; 4 CRCNA UMR S892 INSERM 6299 CNRS Université de Nantes; Centre Hospitalier Universitaire de Nantes, Nantes, F44093 France; 5 Recombinant Antibody Technology Babraham Research Campus, Cambridge, CB22 3AT UK; 6 Centre Hospitalier Universitaire de Bordeaux Laboratoire d’Immunologie et Immunogénétique Hôpital Pellegrin Bordeaux, Bordeaux, F33076 France; 7 Université de Bordeaux UMR CNRS 5164 , Talence, F33400 France; 8 Ligand Pharmaceuticals, San Diego, CA USA

**Keywords:** Humanized rats, Human antibodies, Tetramers, pMHC, Cytofluorimetry

## Abstract

**Background:**

There is an ever-increasing need of monoclonal antibodies (mAbs) for biomedical applications and fully human binders are particularly desirable due to their reduced immunogenicity in patients. We have applied a strategy for the isolation of antigen-specific B cells using tetramerized proteins and single-cell sorting followed by reconstruction of human mAbs by RT-PCR and expression cloning.

**Results:**

This strategy, using human peripheral blood B cells, enabled the production of low affinity human mAbs against major histocompatibility complex molecules loaded with peptides (pMHC). We then implemented this technology using human immunoglobulin transgenic rats, which after immunization with an antigen of interest express high affinity-matured antibodies with human idiotypes. Using rapid immunization, followed by tetramer-based B-cell sorting and expression cloning, we generated several fully humanized mAbs with strong affinities, which could discriminate between highly homologous proteins (eg. different pMHC complexes).

**Conclusions:**

Therefore, we describe a versatile and more effective approach as compared to hybridoma generation or phage or yeast display technologies for the generation of highly specific and discriminative fully human mAbs that could be useful both for basic research and immunotherapeutic purposes.

**Electronic supplementary material:**

The online version of this article (doi:10.1186/s12896-016-0322-5) contains supplementary material, which is available to authorized users.

## Background

Clinical use of monoclonal antibodies (mAbs) to treat autoimmune diseases, transplantation and cancer is having a tremendous medical impact [1]. More than 40 mAbs have been approved for clinical use in the United States and Europe and a large number are currently in development [2, 3]. Initially, mAbs were produced by the immunization of laboratory animals, principally mice and rats. Human recipient immune response against murine mAbs is an important obstacle to their use due to their rapid clearance [4, 5]. To solve this problem, several strategies have been developed including the modification of antibody protein sequences to decrease immunogenicity, such as generation of chimeric mouse-human or humanized antibodies, However, these strategies increase the cost of production and often decrease their affinity [6]. One solution is to generate human mAbs and several strategies are available. One of them is to use human B or plasma cells [7, 8], however this technique is restricted to antigens, such as infectious agents following natural infection, and excludes many important targets that are either normal constituents of the organisms and for which there is immune tolerance or antigens that are harmful if administered, such as toxins. Another technique is the use of phage or yeast display but this generates antibodies with weak affinities, and strategies to increase affinity are costly, time consuming and not always successful. A more recent and effective technique is the use of transgenic animals for human immunoglobulin genes and in which their endogenous immunoglobulin genes are deleted [9]. These immunoglobulin humanized animals can then be immunized with human proteins since their T and B cells will not be tolerant towards these antigens and human antibodies are produced through normal immune responses. The majority of the human mAbs approved for therapy in recent years have been generated in human immunoglobulin transgenic mice [10] but other immunoglobulin humanized transgenic animals, including rats [11–13] and cattle [14] have been described. Overall, current efforts have focused on the use of human mAbs that have reduced immunogenicity after injection in humans compared to chimeric or murine antibodies.

Recently developed human immunoglobulin transgenic animals, such as the rats used in this study [11–13], do not express rat immunoglobulins following genome editing using zinc-finger nucleases and express chimeric immunoglobulin molecules with human antibody recognizing domains and constant regions of rat origin. This allows optimal interaction of cell membrane immunoglobulin receptors with other components of the B-cell receptor (BCR), with generation of antibodies of optimal affinity and diversity displaying extensive mutational changes that accumulate even in rapid immunization schemes. At the same time, it is easy to clone the human antibody sequences in expression vectors containing human constant regions and therefore obtaining fully human antibodies.

Until now, all human mAbs from mouse or rat human immunoglobulin transgenic animals have been generated using the classical hybridoma fusion of total B cells with a myeloma cell line. It results in low frequency of B cell fusing with the myeloma and is followed by intensive cell culture and screening of many cell clones. The procedure is even more complicated when an antibody able to discriminate between highly homologous proteins is required. Thus, the technique of hybridoma generation is time consuming as well as costly and there is need for techniques that will increase efficiency of mAbs generation.

In this study, we describe a procedure allowing selection and isolation of single antigen-specific B cells from a heterogeneous population of B cells based on the use of three color tetramerized antigens (Ags), previously used for the isolation of peptide-major histocompatibility (pMHC) specific T cells [15–17]. We demonstrate the presence of naturally circulating pMHC-specific B lymphocytes in all human peripheral blood samples tested and generated a human mAb against the HLA-A2/Pp65_495_ peptide complex derived from human CMV (hereafter referred as to Pp65) but that displayed of low affinity. We extended this strategy to human immunoglobulin transgenic rats and show that a rapid immunization method [11, 12], followed by single antigen-specific B cell sorting and expression cloning, allowed to obtain fully human IgG high affinity mAbs against four different antigens in ~6–8 weeks. The mAbs, obtained from rats immunized with soluble HLA-A2/Pp65 complex showed increased affinity compared to the one produced from human peripheral blood lymphocytes (PBL) while conserving high peptide discriminative properties. Furthermore, from two animals immunized with human CD22, it was possible to generate at least 27 human mAbs. Thus, the technique of antigen-specific B cell sorting from immunoglobulin humanized rats allowed efficient generation of human mAbs with high affinity and discriminative capacity, likely against any desired antigen. This tetramer-based antigen-specific B cell sorting technique is a faster and more versatile alternative to the use of hybridoma fusion and could also be used in other human immunoglobulin transgenic species.

## Methods

### Donor samples

Cytapheresis samples were obtained from donors seronegative for HCMV, HCV, and HIV and presumably not at risk for infection and with no melanoma (Etablissement Français du sang, EFS, Nantes).

### Animals and immunisation

Human immunoglobulin transgenic rats were rendered deficient for rat heavy, lambda and kappa chain expression using zinc-finger nuclease technology [11, 13] and were made transgenic for human VH, DH and JH gene segments linked to the rat heavy chain region as well as fully human light kappa and lambda chains. These rats expressed a diversified repertoire of antibodies with full human idiotypes [11, 12]. Experimental protocols were approved by the French regional ethical committee for animal experimentation. Following a method previously described that yielded high affinity antibodies [18], human immunoglobulin transgenic rats were immunized at day 0 at each side of the tail base (intra muscular injection) with 100 μg of protein in Complete Freund Adjuvant (CFA) and at day 16 with 100 μg of protein in PBS, then lymph nodes and spleens were harvested at day 21.

### Proteins and pMHC multimers

The following proteins were biotinylated with a DSB-X™ Biotin Protein Labeling Kit (ThermoFisher Scientific): β-Galactosidase (Roche), CD22-Fc (BioTechne), CD22 (ProSci Inc) and Ovalbumin (Roche). The HLA-A*0201–restricted peptides Pp65_495_ (human CMV [HCMV], NLVPMVATV), MelA_27_ (melanoma Ag, ELAGIGILTV), NS3_1073_ (hepatitis C virus [HCV], CINGVCWTV), GagP17_77_ (human Immunodeficiency Virus [HIV], SLYNTVAT) were purchased from GL Biochem (Shanghaï, China). Soluble pMHC monomers were synthesized as previously described [19] HLA-A*0201 H chains used in this study carried a mutation in the α3 domain (A245V), that reduces CD8 binding to MHC class I. Biotinylated proteins and pMHC monomers were tetramerized with either Phycoerythrin (PE)- or allophycocyanin (APC)-labeled premium grade streptavidins (Molecular Probes, Thermo Fischer Scientific, ref S21388 and S32362 respectively) or with Brilliant Violet 421 (BV421)-labeled streptavidin (BioLegend, ref 405225) at a molar ratio of 4:1.

### Tetramer-based enrichment protocol and cell sorting

#### Sorting of human specific B cells

Human Peripheral Blood Mononuclear Cells (PBMCs) were obtained by Ficoll density gradient centrifugation (Eurobio^AbCys^) and split for tetramer staining into tubes containing 200x10^6^ cells. Cells were incubated with 200 μL PE-, APC and BV421-conjugated tetramers diluted to 10 μg/mL in PBS plus 2% FBS for 30 min at room temperature and then washed with 15 ml ice-cold sorting buffer (PBS plus 0.5% BSA plus 0.2% EDTA). The tetramer-stained cells were then enriched using anti-PE and APC Ab-coated immunomagnetic beads [20]. The resulting enriched fractions were stained with anti-human CD3-BV510 (BD Biosciences) and anti-CD19-PerCpCy5.5 (BD Biosciences) mAbs. Stained samples were then collected on an ARIA Cell Sorter Cytometer (BD Biosciences) and single CD19^+^ CD3^−^ PE^+^ APC^+^ BV421^−^ tetramer cells were collected in individual PCR tubes containing 10 μL of 1X PBS, 10 mM DTT (Thermo Fischer Scientific) and 10 units of RNAse Out (Thermo Fischer Scientific) per well. Single cells were frozen at -80 °C immediately. The total number of tetramer-positive B cells was divided by the total number of B cells within the starting PMBC sample to calculate the frequency of pMHC-specific B cells. Counting beads (Dako) were used to normalize results.

#### Sorting of rat specific B cells

Spleen and iliac lymph nodes were harvested from immunized or unimmunized human immunoglobulin transgenic rats 21 days after immunization and cell suspensions prepared. Red blood cells were removed by standard erythrocyte lysis. Cells were then stained with anti-rat IgG (IgG1- and IgG2b-FITC, BD Biosciences) and anti-TCR (R7.3-PerCp, BD Biosciences) mAbs for 30 min at 4 °C. Stained cells were then collected on an ARIA Cell Sorter Cytometer and single IgG^+^ R7.3^−^ PE^+^ APC^+^ and BV421^−^ multimer cells were collected in individual PCR tubes as for human specific B cells.

### Single cell RT-PCR, cloning and IgG1 expression from single B cells

#### Protocol for human B cells

The protocol was adapted from Tiller et al. [21] and Osborn et al. [11]. Antigen specific human B cells in the original PCR tubes were lysed by freezing at −80 °C, followed by heating to 65 °C for 2 min. After cooling to 4 °C, total RNA from the lysed single cell was reverse transcribed in a final volume of 20 μL of 1X Superscript Buffer (Thermo Fischer Scientific) containing 0.5 mM dNTP (Thermo Fisher Scientific), 12.5 μg/mL oligod(T) primers (BioLabs), 2.5 μM random hexamers (Thermo Fischer Scientific), 20 units of RNAse Out (Thermo Fischer Scientific) and 200 units of Superscript Reverse Transcriptase (Thermo Fischer Scientific) for 1 h at 50 °C, after an initial step of 5 min at 25 °C allowing random hexamers hybridation. The reaction was stopped by incubation for 3 min at 95 °C. Variable region of the rearranged heavy chain (HC) locus, lambda (LCλ) or kappa (LCκ) light chain loci were next amplified separately from each single cell cDNA by two round of nested PCRs. For each variable segment, the first round of PCR was performed on 3 μL of cDNA at 94 °C for 4 min, 94 °C for 30s, 58 °C for 30s for HC and LCκ (60 °C for LCλ,) and 55 s at 72 °C for 40 cycles followed by a final elongation step at 72 °C for 7 min in 1X GoTaq Buffer (Promega) containing 1.5 mM MgCl_2_ (Promega), 0.25 mM dNTPs (Thermo Fischer Scientific), 2.5 units of GoTaq DNA Polymerase (Promega), and 100nM of primers (see Additional file 1 for primer sequences) in a 40-μL reaction volume. Four μL of the first amplification products were further amplified by a second round of PCR with inner- sense and anti-sense primers complementary to the 5′ and 3′ ends of the VDJ for heavy chain or VJ for light chain segments respectively and containing at each extremity specific restriction enzymes sites for cloning into expression vectors. The second round of PCR consisted of a denaturation step at 94 °C for 4 min and then 40 amplification cycles (30s at 94 °C, 30s at 58 °C for HC and LCκ/60 °C for LCλ, and 45 s at 72 °C) and a final step at 72 °C for 7 min with 1X GoTaq Buffer (Life Technologies), 1.5 mM MgCl_2_ (Life Technologies), 0.25 mM dNTPs (Life Technologies), 2.5 units of GoTaq DNA Polymerase (Life Technologies), and 100nM of primers (see Additional file 1 for primers sequences) in a 40-μL reaction volume. PCR products from each single cell were detected on a 1.5% agarose GelRed gel (Sigma Aldrich).

PCR products from each well were purified by filtration through a NucleoFast 96 PCR plate (Macherey Nagel, ref 743100.10) digested with respective restriction enzymes in a total volume of 40 μL (AgeI-HF and SalI-HF for HC, AgeI-HF and BsiWI for LCκ, AgeI-HF and XhoI for LCλ). Inserts were respectively cloned into human Igγ1, Igκ or Igλ expression vectors (NCBI Genbank accession numbers: FJ475055 IgG-AbVec, FJ475056 Igκ-AbVec and FJ517647 Igλ-AbVec) containing multiple cloning site upstream of human Ig constant regions and kindly provided by P.C. Wilson [7]. Ligation was performed in a total volume of 20 μL with 1 unit of T4 DNA-Ligase (Thermo Fischer Scientific), 5 μL of digested and purified PCR product and 100 ng of linearized vector. Electrocompetent E. Coli TOP10 bacteria were transformed with 1 μl of the ligation products. Colonies were screened by PCRs using 5′Absense as the forward primer and 3′HuIgG, 3′Cκ494 or 3′Cλ as the reverse primer, respectively. The expected insert band is approximately 400 bp in length. To ensure a consensus variable gene sequence was identified, for each plate, plasmid DNAs from four positive colonies were isolated using Nucleospin Plasmid Purification Kit (Macherey-Nagel) according to the manufacturer’s recommendations and sequenced. Sequences were analyzed by IMGT/V-quest (http://www.imgt.org/IMGT_vquest/share/textes/) to identify germline V(D)J gene segments with highest identity and somatic mutations.

Human embryonic kidney 293A cells were grown as adherent monolayers in DMEM supplemented with 10% FBS and seeded the day earlier in order to reach 70% cell confluency on the day of the transfection. Transient transfections were performed using jetPEI™ (PolyPlus transfection) at a ratio of 1 μg DNA (either 0.5 μg HC and 0.5 μg LC expression vector DNA) to 2 μL jetPEI™ transfection reagent in a total volume of 100 μL in NaCl. The media was removed and the transfection mix added directly to the 293A cells, and 10% FBS DMEM (Thermo Fischer Scientific) was added 2 h later. Cells were washed after 16 h and cultured for 5 days in DMEM supplemented with 1% Nutridoma-SP (Roche). Supernatants were then harvested 5 days later and the secreted mAbs were purified on ProteinA-coated columns and measured by the Recombinant Protein Platform (François Bonamy Society) for MHC or pMHC specific mAbs.

#### Protocol for rat B cells

Single rat B cells in each sorting well received 50 μL of lysis/binding buffer (100 mM TrisHCl (pH 7.5), 0.5 M LiCl, 10 mM EDTA, 1% LiDS, 5 mM DTT) and 40 μg of Dynabeads-oligo(dT)25 (Life Technologies). After 3-5 min, the beads were washed with 100 μL of Wash buffer A (10 mM TrisHCl (pH7.5), 0.15 M LiCl, 1 mM EDTA, 0.1% LiDS) followed by two 50 μL washes with Wash buffer B (10 mM TrisHCl (pH7.5), 0.15 M LiCl, 1 mM EDTA). A final wash using 1x First-strand buffer was carried out before setting up a cDNA synthesis reaction using 50U of Superscript III reverse transcriptase (Life Technologies) and 10U of rRNasin RNase inhibitor (Promega) in a 10 μL reaction volume. After incubation (50 °C, 45 min), the beads were washed with 10 mM Tris-HCl (pH 8.5). A first round PCR was set up in a 30 μL volume of GoTaq Green Master mix (Promega) containing 50nM of each VH and Vκ outer primer and 130nM of both CγCH2 and Cκ outer primers (see Additional file 1 for primer sequences). PCR was performed for 38 cycles with an annealing temperature of 59 °C and an extension of 1 min. A second round PCR of 32-36 cycles was carried out as described above, using 1 μL of the first round as template and either VH inner primers plus CγCH1 or Vκ inner primers and Cκ inner (see Additional file 1 for primer sequences). The PCR products were then sequenced.

Mutated sequences obtained by single RT-PCR were synthesized and cloned in expression vector by GeneArt™ Services at Thermo Fisher Scientific.

Transfection was performed using 2.5 μg of total DNA (1.25 μg heavy chain plasmids + 1.25 μg light chain plasmids). On the day of transfection 2.5 μg of plasmid DNA were diluted in 250 μL of Opti-MEM I medium (Thermo Fisher Scientific) and 10 μl of Lipofectamine (Thermo Fisher Scientific) were diluted in 250 μl of Opti-MEM I medium and incubated at RT for 5 min. DNA was then added to the Lipofectamine mixture and incubated at RT for 20 min. Then the DNA-Lipofectamine complexes were added to each well. Cells were incubated for 5 days, and the supernatants were collected.

### CD22 and β-Galactosidase ELISA

Wells of Maxisorp 96 well flat bottom (Nunc) plates were coated (O/N, 4 °C) with 5 μg/mL of CD22 recombinant protein (ProSci-Inc) or β − Galactosidase protein (Roche) in 50 μl of PBS and were then blocked with 5% BSA in PBS for 2 h at RT. Fifty μL of supernatant were loaded in duplicates wells and incubated for 2 h at 37 °C, followed by the addition of 50 μL of anti human IgG biotinylated antibody (1 μg/mL, Jackson Immunoresearch) for an additional 90 min at 37 °C. HRP-conjugated streptavidin (Jackson Immunoresearch, 1:5000) was added (45 min at 37 °C) and the reaction was visualized by the addition of 50 μL chromogenic substrate (TMB, BD biosciences) for 20 min. The reaction was stopped with 50 μl H_2_SO_4_ and absorbance at 450 nm was measured with reduction at 630 nm using an ELISA plate reader. Plates were washed three times with washing buffer (PBS, pH 7.4, containing 0.5% (v/v) Tween 20) after each step.

### pMHC ELISA

Different monomers were coated O/N at 4 °C in 100 μL of reconstituted ELISA/ELISPOT coating buffer 1X (Affymetrix) at a final concentration of 2 μg/mL in 96-well ELISA plates (Maxisorp, Nunc). Wells were blocked with 10% FBS DMEM medium (Thermo Fischer Scientific) for 2 h at 37 °C. MAbs generated from human PBMC were not purified prior to testing by ELISA and therefore 50 μL of supernatants of transfected 293A cells were added 2 h at RT. MAbs generated from human immunoglobulin transgenic rats immunized with HLA-A2/Pp65 were previously purified and added at 0.5 or 5 μg/mL in 50 μL of PBS for 2 h at RT. An anti-human IgG-HRP Ab (BD Biosciences) was used for detection at 1 μg/mL and incubated for 1 h at RT. The reaction was visualized by the addition of 50 μL chromogenic substrate (TMB, BD biosciences) for 20 min. OD were read at 450 nm.

### Surface plasmon resonance

Surface Plasmon Resonance (SPR) experiments were performed at 25 °C on a Biacore 3000 apparatus (GE Healthcare Life Sciences, Uppsala, Sweden) on CM5 sensorchips (GE Healthcare). Capture mAbs were immobilized at 10 μg/mL by amine coupling using a mixture of N-hydroxysuccinimide and N-ethyl-N′-dimethylaminopropyl carbodiimide, according to the manufacturer’s instructions (GE Healthcare), after a 20-fold dilution in sodium acetate buffer (10 mM, pH 5). Then, ethanolamine (1 M, pH 8.5, GE Healthcare) was injected to deactivate the sensor chip surface. Purified HLA-A*02:01 containing the Pp65_495_ peptide were injected at various dilutions over the capture antibodies 180 s at 40 μL/min. A flow cell left blank was used for referencing of the sensorgrams.

### Anti–HLA antibody testing (Luminex)

The specificity analysis of mAb 5.6 was performed using Single Antigen Flow Bead assays according to the manufacturer’s protocol (LabScreen single-antigen LS1A04, One Lambda, Inc., Canoga Park, CA), exploring 97 class I alleles. The fluorescence of each bead was detected by a Luminex 100 analyser (Luminex, Austin, TX), and recorded as the mean fluorescence intensity (MFI). The positivity threshold for the bead MFI was set at 1000 after removal of the background as previously reported [22]. Clinical relevance of pre-transplant donor-specific HLA antibodies was detected by single-antigen flow-beads.

### Staining experiments on HLA-A2^+^ cells

#### On T2 cells

One million Tap-deficient HLA-A2^+^ T2 cells were loaded for 4 h at 37 °C with specific peptides at 50 μg/mL in 1 mL of 2% FBS RPMI medium (Thermo Fischer Scientific). Cells were then washed with 10 mL PBS, harvested and 1 × 10^5^ cells were stained with purified test mAbs at 1 μg/mL in 200 μL of PBS 2%FBS for 30 min at 4 °C. A secondary goat anti-human IgG-PE Ab (Abcam, ref Ab98596) was added at 1 μg/mL in 50 μL for 30 min at 4 °C. Unloaded T2 or T2 loaded with irrelevant peptides were stained under the same conditions with the same mAbs as controls. Cells were analyzed on a BD FACS Canto II cytometer.

#### On CMV-infected MR5-cells

HLA-A2^+^ MRC-5 cells (RD-Biotech, Besançon, France) were infected at a MOI = 0.1 with human cytomegalovirus (CMV) (Toledo strain) or left uninfected (control). Cells were stained 72 h post-infection with test purified mAb 1.5 at 2 or 10 μg/mL, 30 min at 4 °C in PBS, 0.1% BSA. A secondary PE-conjugated Ab directed against human IgGs (Abcam, ref Ab98596) was added at 1 μg/mL in 50 μL for 30 min at 4 °C. After washing, cells were resuspended in 300 μL PBS and analyzed on a LSR II flow cytometer (BD Biosciences).

#### Blocking experiments on T cell lines

We previously generated, in the lab CRCNA UMR S892, CD8^+^ T cell lines or clones specific to the A2/Pp65 cMelA specific/MelA specific CD8^+^ T cell lines [16]. 1 × 10^5^ T2 cells were loaded O/N at 37 °C in 200 μL of 10%FBS RPMI medium in 96-well round bottom plates with Pp65 (10 pg/mL or 1 ng/mL) or MelA (1 μg/mL) peptides. After washing of target cells, 1 × 10^5^ T cells were incubated with T2 cells in the presence of 10, 50 or 100 μg/mL of isotype control mAb, purified mAbs to be tested or positive control mAbs (anti-HLA-A2 (clone BB7.2), anti-Pan ClassI (clone G46-2.6); BD Biosciences) and in the presence of anti-CD107a/b-FITC mAbs (BD Biosciences), monensin (100nM) and brefeldinA (10 μg/mL) for 4 h at 37 °C in a total volume of 100 μL. Cells were then stained with anti-CD3-APC H7 (BD Biosciences), fixed and permeabilized with 100 μL of Fix/Perm Buffer 1X (Affymetrix) and stained with anti-IFNγ-PE (BD Biosciences) in PermBuffer 1X (Affymetrix). Cells were analyzed on a BD FACS Canto HTS.

## Results

### Detection of pMHC-specific human B cells from healthy human donors

**Fig. 1:**
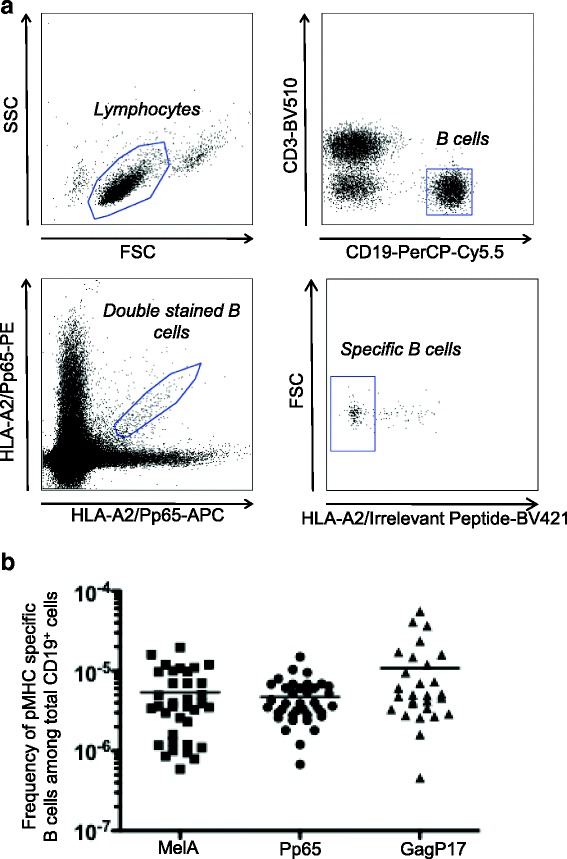
Detection of HLA-A2/Pp65 specific B cells from human PBMC. **a** Gating strategy for HLA-A2/Pp65 specific B cells. After immunomagnetic enrichment with HLA-A2/Pp65 tetramers and staining with additional fluorescent mAbs, cells were analyzed by flow cytometry. Cells were gated first on viable singlet lymphocytes, then on CD19^+^CD3^−^ cells. B cells that stained with both HLA-A2/Pp65-PE and HLA-A2/Pp65-APC tetramers were gated. Cells were also stained with HLA-A2/irrelevant peptides to exclude B cells that were not against HLA-A2/Pp65. **b** The frequency of HLA-A2/MelA (*n* = 36), HLA-A2/Pp65 (*n* = 37), HLA-A2/GagP17 (*n* = 28) specific B cells was determined among total peripheral B cells in healthy donors

**Fig. 2:**
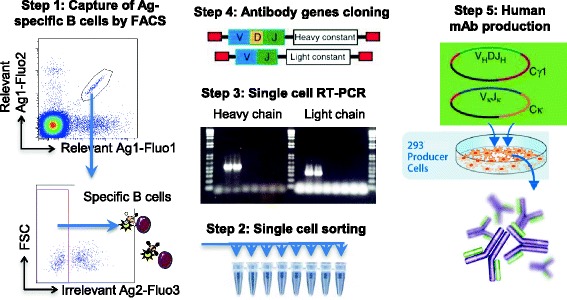
Overall strategy of reconstruction of recombinant human mAbs. A tetramer-based sorting strategy allows detection of B cells of interest. Tetramer^+^ B cells were single cell sorted. Light and heavy chain variable encoding segments were amplified using RT-PCR. Antibody genes PCR products were sequenced directly and cloned into separate eukaryotic expression vectors in frame with constant light and heavy regions genes. Expression of the corresponding fully human mAbs by transiently-transfected HEK 293 cells was purified from the culture supernatant

**Fig. 3:**
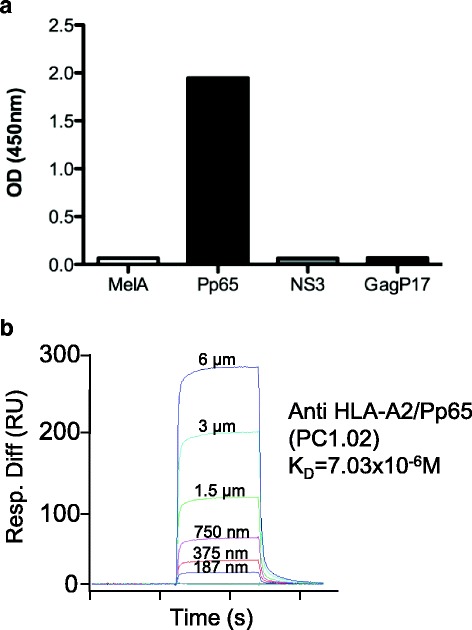
Characterization of a HLA-A2/Pp65 specific mAb (PC1.02) isolated from human peripheral blood. **a** Specificity of mAbs by ELISA. Plates were coated with relevant (HLA-A2/Pp65) or irrelevant HLA-A2 complexes containing HLA-A2-restricted peptides: MelA, NS3 (HCV-1) and GagP17 (HIV-1) at 2 μg/mL, the mAb PC1.02 was added and an anti-human IgG-HRP Ab was used for detection. OD were read at 450 nm. **b** Affinity determination of the mAb PC1.02 using SPR by flowing various concentration of A2/Pp65 complexes over CM5 chip-bound mAb

For the detection of human B cells specific for pMHC tetramers, we adapted a tetramer-based sorting strategy described to measure the frequency of naive CD8^+^ T cells directed against various HLA-A*0201(HLA-A2)/peptide complexes in human healthy individuals [15–17]. Quite surprisingly, during these analyses, we observed a low but sizable fraction of CD3^−^CD19^+^ cells brightly stained by the various HLA-A2/peptide tetramers tested. These included HLA-A2/Pp65_495,_ MelA_27_ derived from melanoma antigen (Ag) and GagP17_77_ derived from HIV, suggesting that B cells expressing specific-BCR might exist in the peripheral blood of healthy donors. The enumeration of these pMHC tetramer^+^ B cells was assessed in a cohort of 20 healthy donors after gating on cells positive for CD19 and negative for CD3, CD14, CD16 and 7AAD. Relevant tetramers with two different flurochromes were used in order to efficiently exclude non-Ag-specific B cell binders presumably recognizing flurochromes as shown in Fig. 1a. Importantly, an irrelevant tetramer associated with a third flurochrome was used to enable exclusion of B cells that could not discriminate the targeted Ag (anti-β2 microglobulin or anti-HLA-A2 complexed or not with other peptides or anti-biotin). Average frequencies of antigen specific cells within B lymphocyte pools were in the range of 5 × 10^−6^-1 × 10^−5^ irrespective of the peptide presented in the binding groove of HLA-A2 (Fig. 1b) and the large majority were IgM^+^IgG^−^ (data not shown). Proper specificity of tetramer^+^ B cells was then assessed through single cell sorting of rare B cells targeting HLA-A2/Pp65 without any previous expansion from one donor, followed by a reconstruction of the antibody expressed by RT-PCR cloning. Using this strategy, we were able to successfully produce one HLA-A2/Pp65 specific antibody starting from 3 sorted single B cells for which Ab-reconstruction was successfully performed (Fig. 2 and data not shown). Although ELISA assays clearly demonstrated this mAb discriminated Pp65 peptide presented by HLA-A2 from other peptides (Fig. 3a), it was not able to recognize HLA-A2^+^ target cells exogenously loaded with a relevant peptide (data not shown). Furthermore, its binding affinity, determined by surface plasmon resonance (SPR), was low (7 × 10^−6^M) (Fig. 3b). We thus described a sensitive protocol allowing detection of even scarce Ag-specific B cells and the generation of highly discriminative human mAbs starting directly from human PBL. Nevertheless, the fact that isolation was performed from non-immune healthy donors and most tetramer^+^ B cells were IgM^+^IgG^−^ likely reduces chances to obtain good Ag-binders. We therefore implemented our strategy to an immunoglobulin humanized rat previously described for the generation of human mAbs by the hybridoma fusion technique [11].

### Tetramer-based isolation of antigen-specific B cells from human immunoglobulin transgenic rats

For the generation of human mAbs in transgenic rats, we deliberately selected antigens of variable difficulty: notably, mAbs that could discriminate between HLA alleles and different peptides loaded on the same allele, such as HLA-A2 and HLA-A2 loaded with Pp65. In this last case, a specific antibody has indeed to recognize both the peptide and the MHC molecule and to discriminate the peptide in the MHC binding groove. We also selected two other antigens, human CD22 and bacterial β-galactosidase (β-gal).

Rats were immunized with these purified reagents and lymph nodes tested for the presence of Ag-specific B cells. Oligomerization of Ags was performed using fluorescently tagged streptavidins for pMHC complexes, CD22 and β-gal proteins. All fluorescently labeled complexes were used with a set of mAbs for identification of IgG^+^ B cells.

**Fig. 4:**
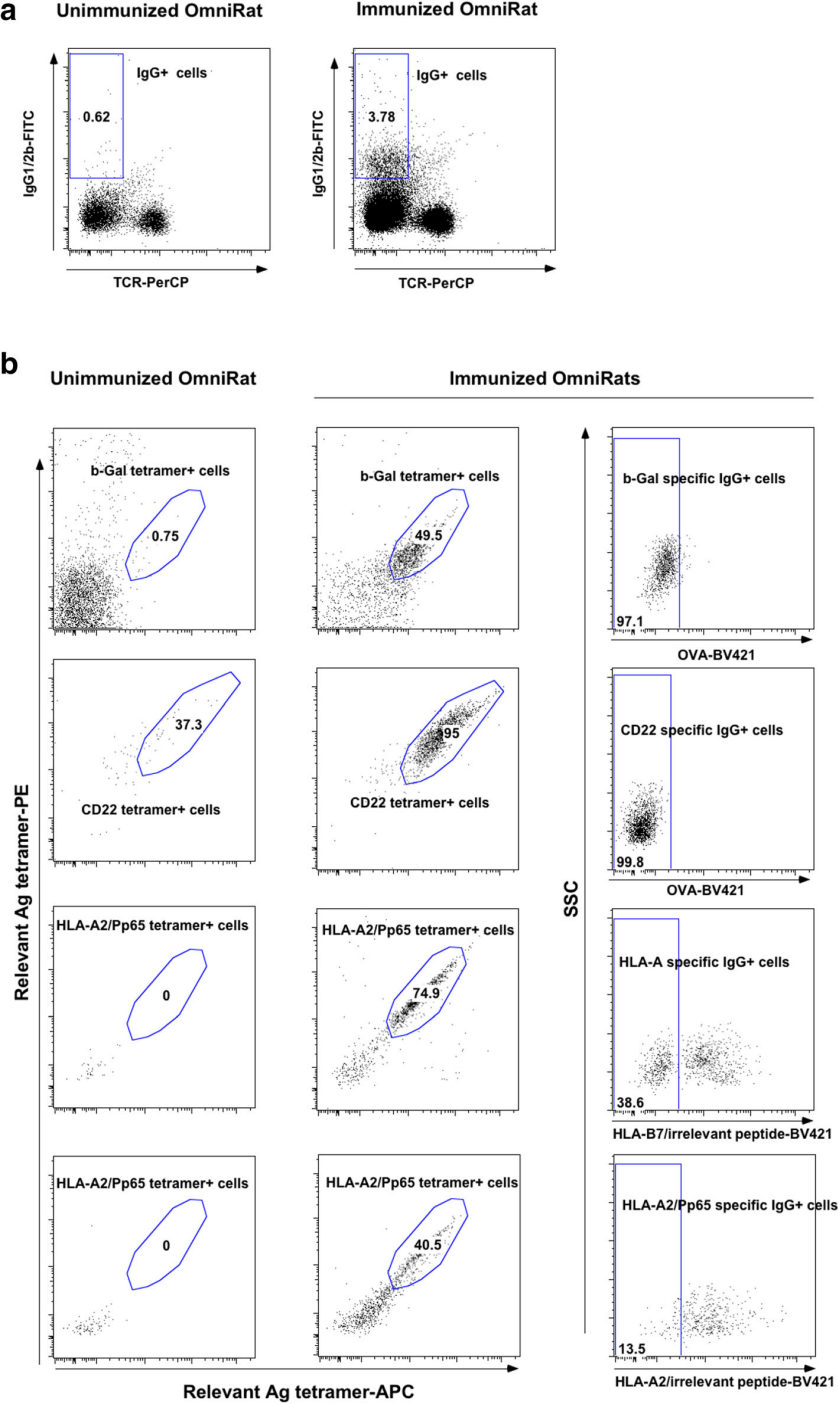
Detection and isolation of Ag-specific IgG1/2b^+^B cells from immunized human immunoglobulin transgenic rats. After immunomagnetic enrichment with Ag tetramers and staining with fluorescent mAbs, cells were analyzed on flow cell sorter. **a** Dots plots show viable and singlets lymphocytes stained with anti-IgG1/2b and anti-TCR (clone R7.3). MAbs from one unimmunized and one immunized human immunoglobulin transgenic rats is shown. **b** Dots plots show B cells stained with relevant Ag tetramers on unimmunized (*left*) and immunized (*right*) human immunoglobulin transgenic with β-Gal, CD22 and HLA-A2/Pp65 Ags. Cells were also stained with irrelevant Ag tetramers to exclude unspecific B cells. Cells from human immunoglobulin transgenic rats immunized with β-Gal and CD22 were stained with ovalbumin tetramers to exclude streptavidin specific B cells. Cells from human immunoglobulin transgenic rats immunized with HLA-A2/Pp65 were stained with HLA-B7/irrelevant peptide tetramers to exclude streptavidin and pan class I MHC-specific B cells, or with HLA-A2/irrelevant peptide tetramers to exclude streptavidin and HLA-A2 specific B cells

Detection and isolation of Ag-specific B cells from transgenic rats by cytometry was similar to the one used for human samples shown in Fig. 1, except the use of an anti-TCR (clone R7.3) antibody for T cell-exclusion and anti-IgG1 and 2b antibodies for positive selection of IgG-expressing B cells (locus γ2a was absent in the transgenic construct [11]). These latter cells were more abundant in immunized transgenic rats than in naive ones (a 5-6 fold increase on average) (Fig. 4a). IgG1/2b^+^ B cells stained by relevant tetramers were detected after immunization of rats with all the antigens tested while these B cells were not detectable in non-immunized animals (Fig. 4b). Once again, the relevant antigens were, labeled with two different fluorochromes and used in association with an irrelevant antigen labeled with a third fluorochrome enabling exclusion of non-antigen specific B cells. This exclusion was performed using an irrelevant tetramer of ovalbumin (OVA) protein for the detection of β-gal and CD22 specific B cells, a HLA-B7/irrelevant peptide complex for the detection of HLA-A2 specific B cells and a mix of HLA-A2/irrelevant peptides complexes for the detection of anti-HLA-A2/Pp65 specific B cells. As expected, this last exclusion strategy was very important as the desired discriminative property of the B cells for the Ag increases. In particular, selection of B cells able to discriminate the HLA-A2/Pp65 complex specifically required this negative selection strategy (Fig. 4b, panel 4) as we observed that most B cells stained by the HLA-A2/Pp65 complex were also able to recognize peptide-loaded HLA-A2 complexes, most-likely because they recognized β2-microglobulin or HLA-A2 specific-epitopes. The tetramer-based strategy thus allows a precise identification of Ag-specific B cells from immunized animals and an initial screening for B cells capable of discriminating between highly homologous proteins before expression cloning and production of specific mAbs as depicted in Fig. 2.

### Binding properties of human mAbs from single B cells

**Fig. 5:**
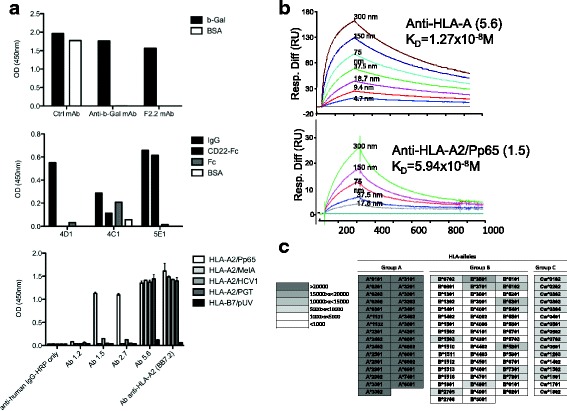
Specificity and affinity of mAbs generated from human immunoglobulin transgenic rats single B cells. **a** Specificity ELISA of mAbs generated from human immunoglobulin transgenic rats immunized with β-Gal (*upper panel*), CD22 (*middle panel*) and HLA-A2/Pp65 (*lower panel*). Upper panel: a representative anti-β-Gal specific mAb (F2.2) among those generated, a control mAb that nonspecifically binds to different proteins (Ctrl mAb) and a positive control human anti-β-Gal mAb previously generated by us [11]. Middle panel: 4D1 mAb did not recognize any epitope on CD22-Fc fusion-protein, 4C1 mAb recognized an epitope on the Fc domain of the fusion-protein, whereas 5E1mAb specifically recognized the CD22 protein. Lower panel: mAbs 1.5 and 2.7 recognized only the HLA-A2/Pp65 monomer, mAb 5.6 recognized all monomers containing the HLA-A2 molecule and anti-HLA-A2 mAb clone BB7.2 was added as a positive control. **b** Determination of the affinity of mAbs 5.6 and 1.5 generated from human immunoglobulin transgenic rats immunized with HLA-A2/Pp65 using surface plasmon resonance by flowing various concentration of HLA-A2/Pp65 complexes over CM5 chip-bound mAb. **c** Analysis of the specificity of mAb 5.6 in a Luminex single antigen bead assay. Results are shown in terms of interval MFI. Positivity threshold was set at 1000

Single IgG1/2b^+^ B cells were isolated for every Ag tested and specific mAbs were obtained in every instance (Table 1). The proper specificity of these mAbs was analyzed and results show exquisite specificity for their respective ligands such as discrimination between the Fc and CD22 moieties of the Fc-CD22 molecule used for immunization and between pMHC in a peptide-dependent manner (Fig. 5a). Affinity was measured by SPR and all antibodies showed high binding affinities: anti-β-gal mAb, K_D_ = 1 × 10^−10^ to 1 × 10^−7^M; anti-CD22, K_D_ = 1.6 × 10^−9^M; anti-HLA-A2 mAb, K_D_ = 1.27 × 10^−8^M; anti-HLA-A2/Pp65 = K_D_: 1.44 × 10^−7^M and 5.94 × 10^−8^M (Fig. 5b, Table 1). In the latter case, a 2 log fold increase in affinity was observed between HLA-A2/Pp65 specific mAbs generated from humanized rats versus human B cells, highlighting the efficiency of the humanized rat system.

**Table 1:** Analysis of isolated antigen-specific B cells

Targeted antigen	Number of analyzed wells	Number of wells with HC gamma amplification	Number of wells with HC and LC associated (% recovery)	Mutations in variable HC and LC	Number of antigen-specific produced antibodies	Affinity determined by SPR (K_D_)
β-galactosidase	14	12	8 (57%)	Yes	8	1 × 10^−10^ to 1 × 10^−7^M
Fc-CD22	30	6	6 (20%)	Yes	1 anti-CD22; 2 anti-Fc	1,6 × 10^−9^M
HLA-A	7	6	6 (86%)	Yes	1	1,27 × 10^−8^M
HLA-A2/Pp65	24	11	9 (38%)	Yes	3	6.10^−8^ to 1,4.10^−7^M
CD22	72	49	44 (61%)	Yes	27	Not determined

Anti-HLA-A2 and anti-HLA-A2/pPp65 mAbs generated with transgenic rats were further examined in order to evaluate their ability to discriminate specific ligands from other highly related Ags (i.e. other pMHC complexes) in more physiological contexts. The anti-HLA-A2 mAb (clone 5.6) was first tested in a Luminex assay against microbeads loaded with class I antigens. As shown in Fig. 5c, this antibody recognized HLA-A*0201 and all the HLA-A group but was poorly or not reactive against most HLA-B or -C antigens, demonstrating its ability to discriminate between MHC-groups but not between HLA-A alleles. Although B cells after immunization of human immunoglobulin transgenic rats were initially selected for their ability to bind HLA-A*0201 tetramers, this global anti-HLA-A specificity was at least in part expected as the initial exclusion strategy of Ag-specific B cells was performed using HLA-B7 complexes and not other HLA-A alleles. The anti-HLA-A2/Pp65 mAbs (clones 1.5 and 2.7) were not able to recognize any MHC class I loaded beads in Luminex assays (data not shown).

**Fig. 6:**
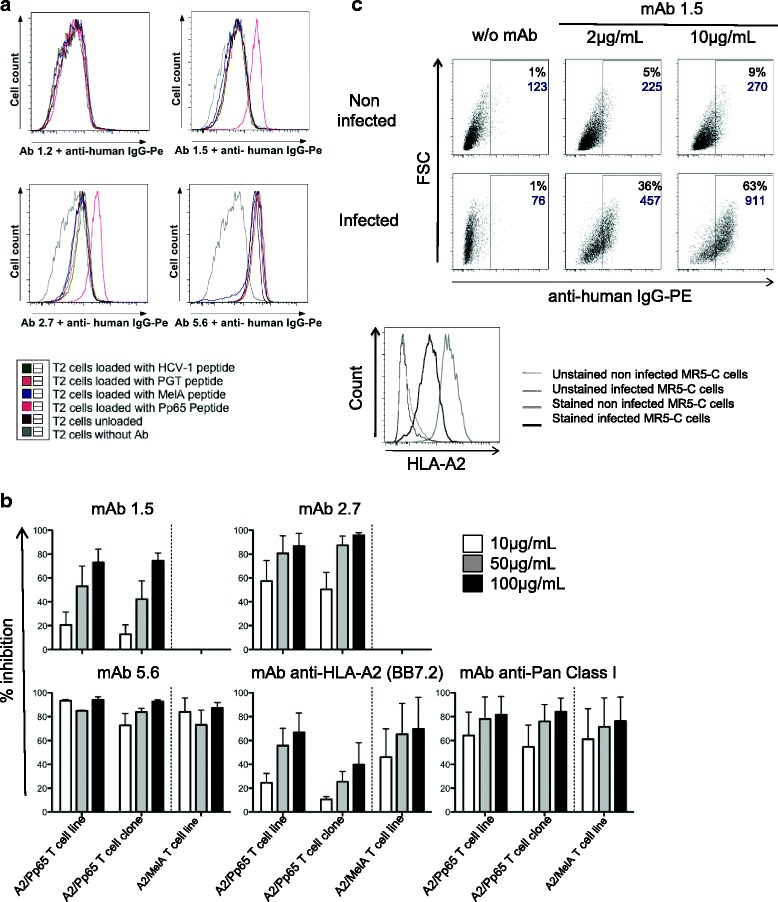
Use of mAbs generated from human immunoglobulin transgenic rats immunized with HLA-A2/Pp65 in functional experiments. **a** T2 cells (HLA-A2^+^) were loaded for 4 h with different peptides at 50 μg/mL or not (unloaded T2 cells), then stained with purified anti-HLA-A or anti-HLA-A2/Pp65 mAbs (clones 1.2, 1.5, 2.7 and 5.6) at 1 μg/mL. A secondary Ab anti-human IgG-PE was added at 1 μg/mL. Cells were analyzed on a BD FACS Canto II cytometer. Specific anti-HLA-A2/Pp65 staining was observed with mAbs 2.7 and 1.5. **b** One A2/Pp65 specific T cell line, one A2/Pp65 specific T cell clone and one A2/MelA specific T cell line were stimulated with T2 cells loaded O/N respectively with Pp65 or MelA peptides in the presence of 10, 50 or 100 μg/mL of isotype control mAb, purified mAbs to be tested or positive control mAbs (anti-HLA-A2 (clone BB7.2), anti-pan ClassI; BD Biosciences). The percent inhibition of T-cell activation is indicated in the figure (read-out: IFNγ production). Three independent experiments were performed. **c** MRC-5 cells infected (MOI = 0,1) or not with CMV virus (Toledo strain) were stained 72 h post-infection with purified mAb 1.5 at 2 or 10 μg/mL or not (MRC-5 cells without Ab). A secondary Ab anti-human IgG-PE was added at 1 μg/mL (upper panel). Percentages of PE^+^ cells are indicated on dot plots in black, as well as the geometric mean of PE fluorescence in purple. Staining with an anti-HLA-A2 mAb (BD Biosciences) was also performed on non-infected (*grey line*) or infected MR-5 cells (black line); dotted lines represents unstained cells respectively (lower panel)

Both anti-HLA-A and anti-HLA-A2/Pp65 mAbs were then tested for their ability to (i) recognize target cells expressing or not HLA-A2 loaded with relevant or irrelevant peptides (ii) inhibit specific-T cell activation in a MHC or pMHC restricted manner and (iii) specifically bind HLA-A or HLA-A2/Pp65 complexes generated under naturally occurring physiological Ag-processing (i.e. viral infection) (Fig. 6). While the anti HLA-A mAb (clone 5.6) was able to recognize HLA-A2^+^ target cells and block activation of HLA-A2 reactive T cell clone or lines, in a MHC group-A dependent manner but irrespective of the loaded-peptide, the anti-HLA-A2/Pp65 mAbs (clones 1.5 and 2.7) were dependent on both the peptide and the expression of HLA-A2 (Fig. 6a and b). Human embryonic fibroblasts, expressing or not HLA-A2, were next infected with the HCMV laboratory strain Toledo at a MOI of 0.1. Seventy-two hours later, a strong decrease in the expression of HLA-A on the surface of the virus-infected cells was observed, in agreement with the previously documented HCMV-induced downregulation of MHC expression (Fig. 6c bottom) [23]. Although the overall MHC-expression on infected cells was strongly affected, the anti-HLA-A2/Pp65 mAb 1.5 was able to stain infected cells in an HLA-A2 dependent manner, demonstrating its ability to recognize a naturally generated viral peptide/MHC ligand (Fig. 6c upper).

Our strategy shows that human immunoglobulin transgenic rats can efficiently generate high affinity human mAbs against all tested antigens, and these are capable of discriminating between highly homologous proteins without cell culture techniques.

### Overall yield recovery of human mAbs from transgenic rats

The initial screening of selected B cells was performed for various antigens but on a limited number of cells. From a total of 75 single isolated B cells (*n* = 14 for β-gal, 30 for CD22-Fc, 7 for HLA-A2, 24 for HLA-A2/Pp65), couples of heavy and light chain segments for a single B cell clone were amplified 50% of the time, irrespective of the targeted Ag (Table 1). Sequence analysis of amplified heavy chain encoding segments confirmed that all purified B cells expressed IgG (Additional file 2: Table S1). We also observed a variety of different VJ or VDJ segments (Additional file 2: Table S1), in agreement with the previously demonstrated ability of humanized rats to produce diverse B cell responses [11]. Most analyzed sequences revealed the presence of somatic hypermutations, indicating that these cells had undergone affinity maturation (Additional file 2: Table S1). Efficient production of corresponding recombinant mAbs was obtained for all specificities with a global recovery yield of 42% (Table 1), but with large variations depending on the Ag-“complexicity”. As an example, 8 anti-β-gal mAbs were generated from 8 couples of heavy and light chains segments (recovery = 100%), while only 3 anti-HLA-A2/Pp65 mAbs were obtained from 9 couples (recovery = 33,3%), underlining the difficulty for these mAbs to recognize both the peptide and the MHC molecule.

**Fig. 7:**
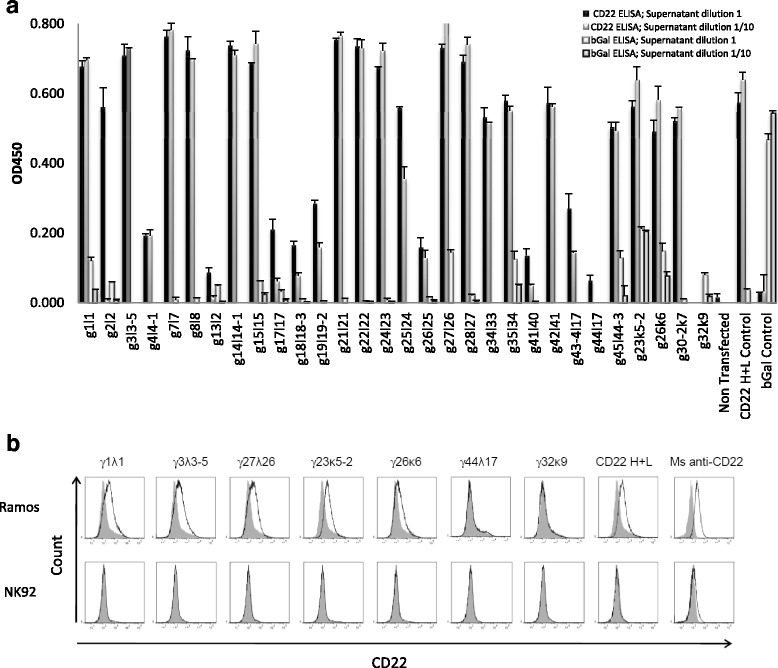
CD22 specificity analysis of antibodies by ELISA and flow cytometry. Supernatants from HEK 293 cells transfected with light and heavy chain coding constructs from single anti-human CD22 B cells were analysed for reactivity against human CD22. **a** Human CD22 or β-Gal were coated at a concentration of 5 μg/mL in 96-well plates. 50 μL of IgG expression supernatants collected 5 days after transfection were used pure or diluted at 1:10 in PBS and used as the primary antibody. Biotin conjugated anti human IgG H + L antibody was used as the secondary antibody. Streptavidin HRP was used for detection. OD450 read from blank control was deducted from all wells. Cell culture supernatants from human anti-β-Gal H + L (clone 44A8, β-Gal Control) and CD22 H + L (clone 5E1, CD22 H + L Control) transfected cells were used as positive controls in β-Gal and CD22 ELISA, respectively. Non-transfected cell culture supernatant was used as the negative control. **b** Representative flow cytometry analysis of heavy and light chain co-transfected HEK cell culture supernatants. Supernatants from the indicated heavy + light chain positive transfections in ELISA (the first 5 from left) or negative (the following ones) and were used as primary antibodies on Ramos CD22^+^ and NK92 CD22^−^ cell lines. Cell culture supernatants from human anti-CD22 H + L (clone 5E1, CD22 H + L) transfected cells and a commercial mouse mAb anti-human CD22 (Ms anti-hu CD22) were used as positive controls. Biotin conjugated anti human IgG H + L antibody was used as a secondary antibody and streptavidin Alexa405 was used for cytometry detection. Histograms for transfected cell supernatant shown in black non-transfected cell supernatant in filled grey. Control Ramos and NK92 cell lines were labelled with a commercial mouse anti human CD22 FITC (*black line*) or with an isotype control (*filled grey*)

Although we were able to obtain Ag-specific mAbs in all cases (Table 1), a deeper analysis of the yield recovery was performed from two rats immunized against human CD22. Three weeks after immunization, the lymph nodes were collected and among IgG1/2b^+^ B lymphocytes, 123 were specifically stained by CD22 tetramers. Single cell RT-PCR was performed on 72 of these B cells resulting in amplification of both heavy and light chains encoding segments in the same B cell in 44 of them (total efficiency of 61%, Table 1). Sequence analysis confirmed the induction of a polyclonal secondary B-cell response in the immunized rat and the presence of many somatic hypermutations (Additional file 3: Table S2). These 44 mAbs were then produced by expression cloning and transfection followed by screening for their ability to recognize CD22 by ELISA and cytometry (Fig. 7a and b). From these 44 paired sequences, 27 specifically recognized CD22 protein by ELISA analysis (Fig. 7a), reacting with different intensities that were not correlated by levels of IgG expression in each transfectant (data not shown). Analysis by cytometry showed that 20 of the 44 mAbs reacted against CD22^+^ target cells and not against CD22^−^ target cells and these were among the 27 depicted by ELISA (Fig. 7b). The absence of CD22 detection by cytometry by some mAbs (7/27) is possibly explained by detection of epitopes presented by denatured CD22 proteins used in the ELISA analysis, not expressed in the natural cell membrane conformation, or due to low antibody affinities. From these 27 mAbs, further analyses of 4 of them randomly chosen showed for all a high affinity (>2.43 nM) and all recognized human blood B cells. An additional file shows this in details (see Additional file 4).

## Discussion

Using multiparametric single cell sorting of highly antigen-specific B cells from the blood of human donors followed by expression cloning of immunoglobulin genes, we generated human mAbs. These mAbs were highly specific since they discriminated between different pMHC complexes. Nevertheless, they were of TCR-like affinity (between 1 and 10 × 10^−6^M). Indeed, most B cells isolated from human blood and against a protein target were naïve (IgM^+^ IgG^−^), in agreement with the fact that human donors were not immunized against these particular Ag. Immunoglobulin humanized transgenic rats produced highly diverse and near normal expression levels of antibodies with human idiotypes [11, 12]. Using a classical long immunization protocol into the footpad resulting mainly in antigen-specific B cells in the spleen followed by the hybridoma technique, we previously showed the generation of human mAbs against several proteins [11]. Although well established, this immunization protocol takes more than 6 weeks and requires administration of 400 μg of antigen [11]. Furthermore, the hybridoma technique is costly and time consuming. Thus, we developed a rapid and efficient immunization protocol allowing rapid production of antigen-specific B cells in the draining lymph nodes followed by the same single cell multiparametric sorting of antigen-specific B cells and expression cloning of immunoglobulin genes used from human PBMCs. In three weeks using 200 μg of antigen, it has been described that high affinity antibodies are generated [18] and we show here that specific B cells could be isolated from medial iliac lymph nodes. Single cell RT-PCR and sequencing of the immunoglobulin genes of these B cells showed a high proportion of mutated IgG sequences with high affinity, regardless of antigen provenance (human or not) and regardless of the size of proteins. This rapid affinity maturation has already been described [24].

The gating strategy to isolate Ag-specific B cells required three colors for antigen labeling (two relevant and one irrelevant or related Ags). Similar techniques using only two colored Ags allowed isolation of antigen-specific B cells from immunized wild type mice (one color for the specific antigen and another one for a nonspecific protein) [25] or from HIV-specific blood B cells from HIV^+^ patients (two color Ag specific labeling) [8] have been described. Compared with the latters, the 3 color gating strategy improved the antigen-specificity of the sorted B cells since the 2 antigen-specific colors allow exclusion of fluorochrome-specific B cells, while the third irrelevant/related Ag allows to gate-out streptavidin and biotin-specific B cells and could offer the possibility to identify B cells able to discriminate between highly related-Ags. This latter point is best exemplified by our ability to generate HLA-A2/Pp65-specific human mAbs from human PBL or humanized rats. In that particular case, the use of an HLA-A2/irrelevant peptide complex is mandatory to eliminate or reduce, biotin, streptavidin, β2-microglobulin, but also HLA-A2 specific B cells that do not discriminate the peptide in the HLA-A2 binding groove. The use of 3 colored Ags has recently been performed to isolate anti-citrullinated-specific B cells from rheumatoid arthritis patients [26] but has not been yet exploited to isolate B cells able to discriminate between related/homologous antigens and to produce corresponding human mAbs from human or animals. One anti-HLA-A2/Pp65 mAb tested recognized the epitope with high affinity (6x10^−8^M) allowing detection in CMV infected cells and thus could be a useful reagent for diagnostic or even therapeutic purposes. This antibody will be tested in the future for its capacity to kill cells by ADCC or by using its antigen-binding domain to generate a chimeric-antigen receptor then expressed by cytotoxic T lymphocytes. It is noteworthy that this HLA-A2/Pp65-specific mAb showed a two log increase affinity compared to the one that we obtain from human PBL and one log over an anti-HLA-A2/Pp65 generated by phage display [23]. These observations underlie the power of human immunoglobulin transgenic animals system where immunization with virtually any human or microbial protein could be easily performed. Moreover, we readily obtained high affinity mAbs (1x10^−8^ to 1x10^−10^M) against the other antigens targeted in our study (human CD22, β-Galactosidase, and HLA-A2).

Previous studies have questioned the suboptimal performance of human immunoglobulin transgenic animals, such in transgenic mice, both in terms of the diversity and affinity of the antibodies generated [10]. Indeed, an imperfect interaction between the constant region of the human immunoglobulin, expressed on the B cell membrane, and the mouse B-cell receptor (BCR) signaling machinery, could decrease the efficacy and quality of antibody production [27]. A successful strategy to overcome this has been to generate transgenic animals carrying a heavy immunoglobulin transgene locus in which human immunoglobulin V_H_, D_H_, and J_H_ segments have been linked to mouse [28] or rat [11] heavy immunoglobulin constant chain loci. In this situation, membrane antibodies with a constant region from the transgenic species interact optimally with other components of the BCR. These transgenic rats also harbor an entirely human immunoglobulin light chain transgene locus. We demonstrate in the present study that B cells from human immunoglobulin transgenic rats express a normal diversity of antibodies with human idiotypes of high affinity against various Ags. These can be easily converted to fully humanized Abs by cloning the variable sequences in an expression vector containing human constant heavy chain sequences [11].

The cell sorting strategy of positively selecting IgG^+^ specific B cells resulted in a high percentage of specific mAbs. For example, following CD22 immunization, 61.4% of IgG^+^ cells were CD22 specific, with β-Gal immunization, 100% of IgG^+^ cells were β-Gal specific and with HLA-A2/Pp65 immunisation, 33.3% of IgG^+^ cells were HLA-A2/Pp65 specific. In comparison, 4 to 14% of total hybridomas are estimated to be Ag specific using the hybridoma technique [29]. Twenty-seven anti-CD22-specific mAbs were obtained using 58% of the total number of antigen-specific B cells from 2 animals. Assuming that all the antigen-specific B cells would have been analyzed from a given animal, a recovery estimate of 20 specific mAbs could be inferred per animal. Compared to hybridoma generation, multiparametric cell sorting of Ag specific B cells followed by single cell RT-PCR analysis, sequencing and cloning in expression plasmids, considerably reduces the time required to isolate specific mAbs by eliminating cell culture to isolate and expand hybridomas. A drawback of our technique is that it relies on the availability of purified antigen to be labelled with biotin and perform B cell sorting. Importantly, the immunization step could be replaced by DNA immunization or using cells expressing a given antigen.

Because plasma cells secrete high affinity antibodies, it would be very desirable to isolate antigen specific plasma cells. By using a reticulum endoplasmic marker and fluorescent antigen staining, Kurosawa et al. demonstrated the feasibility of isolating polyclonal plasma/plasmablast cells by FACS from humans and several wild-type animals [30]. Subsequent expression of heavy and light chains allowed them to produce mAbs. As they demonstrated the capacity of plasma cells isolation from rats, this technique is reasonably foreseeable for transgenic rats. Other techniques have been applied to generate mAbs, such as immortalization of human B cells with Epstein Barr virus [31] but this strategy has drawbacks linked to the use of human B cells described above. Another technique used in immunized animals is next generation sequencing coupled to bioinformatic analysis to pair the more abundant heavy and light chains [32] but this technique needs extensive bioinformatic analysis as well cloning and expression of sequences.

## Conclusions

The use of human immunoglobulin transgenic rats, the rapid immunization followed by an efficient single cell sorting of antigen-specific IgG^+^ B cells, allowed production of human IgG mAbs against all antigens tested, with high yield affinity and discrimination, in a rapid manner. This technique could be easily implemented to other immunoglobulin humanized species and adapted to virtually any desired antigens which should open interesting perspectives both for basic research and immunotherapeutic purposes.

## Additional files

Additional file 1: Primer sequences. Primer sequences for Ag-specific Ig amplification and for CD22-specific Ig (yield recovery of human mAbs). (DOCX 122 kb)
Additional file 2: Table S1. Results of Ag-specific Ig genes sequencing. (DOCX 113 kb)
Additional file 3: Table S2. Results of CD22-specific Ig genes sequencing. (DOCX 141 kb)
Additional file 4: Analysis of anti-human CD22 human mAbs affinity and specificity on human PBMCs. a) Affinity determination of anti-human CD22 mAbs using SPR by flowing various concentration of CD22 antibody over CD22 chip-bound. b) Flow cytometry analysis of CD22 mAbs on human PBMC. Human PBMC were labeled with anti human CD19 (APC) and purified CD22 mAbs (clone γ1λ1, γ3λ3-5, γ23κ5-2 or γ27λ26) coupled with Alexa Fluor 568 fluorochrome or with a commercial mouse mAb anti-human CD22 (Ms anti-human CD22). (PPT 666 kb)

## Abbreviations

β-gal: β-galactosidase
Ag: Antigen
APC: Allophycocyanin
BCR: B-cell receptor
BV: Brillant violet
CFA: Complete Freund Adjuvant
HC: Heavy chain
LC: Light chain
mAbs: Monoclonal antibodies
OVA: Ovalbumine
PBL: Peripheral Blood Lymphocytes
PBMC: Peripheral Blood Mononuclear Cells
PE: Phycoerythrin
pMHC: Peptide-major histocompatibility complex
SPR: Surface Plasmon Resonance
TCR: T-cell receptor
