# Supplementary material for: Antigen-specific single B cell sorting and expression-cloning from immunoglobulin humanized rats: a rapid and versatile method for the generation of high affinity and discriminative human monoclonal antibodies
Source: BMC Biotechnol. 2017 Jan 9;17:3. doi: 10.1186/s12896-016-0322-5 (PMC5234254; doi:10.1186/s12896-016-0322-5)
Supplement: Additional file 1: — Primer sequences. Primer sequences for Ag-specific Ig amplification and for CD22-specific Ig (yield recovery of human mAbs). (DOCX 122 kb) [file 12896_2016_322_MOESM1_ESM.docx]

**Supplemental Material – Primers sequences**

**Primer sequences for Ag-specific Ig amplification**

**Amplification of heavy chains.**

| **Outer primers** | | **Inner primers** | |
| --- | --- | --- | --- |
| ***Forward primers*** | | ***Forward primers*** | |
| 5LVH1 | ACAGGTGCCCACTCCCAGGTGCAG | 5AgeIVH1_5_7 | CTGCAACCGGTGTACATTCCGAGGTGCAGCTGGTGCAG |
| 5LVH3 | AAGGTGTCCAGTGTGARGTGCAG | 5AgeIVH3 | CTGCAACCGGTGTACATTCTGAGGTGCAGCTGGTGGAG |
| 5LVL4_6 | CCCAGATGGGTCCTGTCCCAGGTGCAG | 5AgeIVH3_23 | CTGCAACCGGTGTACATTCTGAGGTGCAGCTGTTGGAG |
| 5LVH5 | CAAGGAGTCTGTTCCGAGGTGCAG | 5AgeIVH4 | CTGCAACCGGTGTACATTCCCAGGTGCAGCTGCAGGAG |
|  |  | 5AgeIVH4_34 | CTGCAACCGGTGTACATTCCCAGGTGCAGCTACAGCAGTG |
| ***Reverse primers for human Ig*** | | 5AgeIVH1_18 | CTGCAACCGGTGTACATTCCCAGGTTCAGCTGGTGCAG |
| 3HuIgG_const_anti | TCTTGTCCACCTTGGTGTTGCT | 5AgeIVH1_24 | CTGCAACCGGTGTACATTCCCAGGTCCAGCTGGTACAG |
| 3CuCH1 | GGGAATTCTCACAGGAGACGA | 5AgeIVH3__9_30_33 | CTGCAACCGGTGTACATTCTGAAGTGCAGCTGGTGGAG |
|  |  | 5AgeIVH6_1 | CTGCAACCGGTGTACATTCCCAGGTACAGCTGCAGCAG |
| ***Reverse primers for OmniRat® Ig*** | | ***Reverse primers*** | |
| Rat γ CH2 | GGGAAGATGAAGACAGATG | 3SalIJH1_2_4_5 | TGCGAAGTCGACGCTGAGGAGACGGTGACCAG |
|  |  | 3SalIJH3 | TGCGAAGTCGACGCTGAAGAGACGGTGACCATTG |
|  |  | 3SalIJH6 | TGCGAAGTCGACGCTGAGGAGACGGTGACCGTG |

**Amplification of ligh chains κ.**

| **Outer primers** | | **Inner primers** | |
| --- | --- | --- | --- |
| ***Forward primers*** | | ***Forward primers*** | |
| 5'LVk1_2 | ATGAGGSTCCCYGCTCAGCTGCTGG | 5'AgeIVk1 | CTGCAACCGGTGTACATTCTGACATCCAGATGACCCAGTC |
| 5'LVk3 | CTCTTCCTCCTGCTACTCTGGCTCCCAG | 5'AgeIVk1_9_1–13 | TTGTGCTGCAACCGGTGTACATTCAGACATCCAGTTGACCCAGTCT |
| 5'LVk4 | ATTTCTCTGTTGCTCTGGATCTCTG | 5'AgeIVk1D_43_1_8 | CTGCAACCGGTGTACATTGTGCCATCCGGATGACCCAGTC |
|  |  | 5'AgeIVk2 | CTGCAACCGGTGTACATGGGGATATTGTGATGACCCAGAC |
| ***Reverse primers*** | | 5'AgeIVk2_28_2_30 | CTGCAACCGGTGTACATGGGGATATTGTGATGACTCAGTC |
| 3'Ck543_566 | GTTTCTCGTAGTCTGCTTTGCTCA | 5'AgeVk3_11_3D_11 | TTGTGCTGCAACCGGTGTACATTCAGAAATTGTGTTGACACAGTC |
|  |  | 5'AgeVk3_15_3D_15 | CTGCAACCGGTGTACATTCAGAAATAGTGATGACGCAGTC |
|  |  | 5'AgeVk3_20_3D_20 | TTGTGCTGCAACCGGTGTACATTCAGAAATTGTGTTGACGCAGTCT |
|  |  | 5'AgeVk4_1 | CTGCAACCGGTGTACATTCGGACATCGTGATGACCCAGTC |
|  |  | ***Reverse primers*** | |
|  |  | 3'BsiWIJk1_2_4 | GCCACCGTACGTTTGATYTCCACCTTGGTC |
|  |  | 3'BsiWIJk3 | GCCACCGTACGTTTGATATCCACTTTGGTC |
|  |  | 3'BsiWIJk5 | GCCACCGTACGTTTAATCTCCAGTCGTGTC |

**Amplification of light chains λ.**

| **Outer primers** | | **Inner primers** | |
| --- | --- | --- | --- |
| ***Forward primers*** | | ***Forward primers*** | |
| 5'LVl1 | GGTCCTGGGCCCAGTCTGTGCTG | 5'AgeIVl1 | CTGCTACCGGTTCCTGGGCCCAGTCTGTGCTGACKCAG |
| 5'LVl2 | GGTCCTGGGCCCAGTCTGCCCTG | 5'AgeIVl2 | CTGCTACCGGTTCCTGGGCCCAGTCTGCCCTGACTCAG |
| 5'LVl3 | GCTCTGTGACCTCCTATGAGCTG | 5'AgeIVl3 | CTGCTACCGGTTCTGTGACCTCCTATGAGCTGACWCAG |
| 5'LVl4_5 | GGTCTCTCTCSCAGCYTGTGCTG | 5'AgeIVl4_5 | CTGCTACCGGTTCTCTCTCSCAGCYTGTGCTGACTCA |
| 5'LVl6 | GTTCTTGGGCCAATTTTATGCTG | 5'AgeIVl6 | CTGCTACCGGTTCTTGGGCCAATTTTATGCTGACTCAG |
| 5'LVl7 | GGTCCAATTCYCAGGCTGTGGTG | 5'AgeIVl8 | CTGCTACCGGTTCCAATTCYCAGRCTGTGGTGACYCAG |
| 5LVl8 | GAGTGGATTCTCAGACTGTGGTG |  |  |
|  |  |  |  |
| ***Reverse primers*** | | ***Reverse primers*** | |
| 3'Cl | CACCAGTGTGGCCTTGTTGGCTTG | 3'XhoICl | CTCCTCACTCGAGGGYGGGAACAGAGTG |

**Sequencing primer**

| Ab-vec-sens | GCTTCGTTAGAACGCGGCTAC |
| --- | --- |

**Primer sequences for CD22-specific Ig (yield recovery of human mAbs)**

**Amplification of heavy chains.**

| **Outer primers** | | **Inner primers** | |
| --- | --- | --- | --- |
| ***Forward primers*** | | ***Forward primers*** | |
| VH1_24 leader | GGACTGCACCTGGAGGATC | VH1, VH7-4 leader in | TCTTCTTGGTGGCAGCAGC |
| VH1_vh7_4 leader | ATGGACTGGACCTGGAGGATC | VH2 in | TCACCTTGAAGGAGTCTGGTCC |
| VH2_5 leader | CATCCCTTCATGGGTCTTGTC | VH3_07 VH3_23 FR1 | AGGTGTCCAGTGTGAGGTGC |
| VH2_26 leader F | GCTACACACTCCTGCTGCTGACC | VH3_09 FR1 | AAGGTGTCCAGTGTGAAGTGC |
| VH3 leader F | ATGGAGTTTGGGCTGAGCTGG | VH3_11 3_30 3_33 FR1 | AGGTGTCCAGTGTCAGGTGC |
| VH3_07 leader F | ATGGAATTGGGGCTGAGCTG | VH4 in | GCTCCCAGATGGGTCCTG |
| VH3_09 leader F | ATGGAGTTGGGACTGAGCTGGA | VH6 in | ATCTTCCTGCCCGTGCTG |
| VH4 leader F | TGAAACACCTGTGGTTCTTCC |  |  |
| VH4_39 leader F | TGAAGCACCTGTGGTTCTTCC |  |  |
| VH6 leader out | ACAATGTCTGTCTCCTTCCTCATC |  |  |
| ***Reverse primers*** | | ***Reverse primers*** | |
| γ CH2 R | CTTTGGSGGGAAGATGAAGACAGATG | γ CH1 R | GGACAGGGCTCCAGAGTTCC |

**Amplification of light chains κ.**

| **Outer primers** | | **Inner primers** | |
| --- | --- | --- | --- |
| ***Forward primers*** | | ***Forward primers*** | |
| hs VK1 leader | ATGAGGGTCCCCGCTCAG | hs_Vk1_lead_in | TCAGCTCCTGGGGCTCCT |
| hs VK1_16 leader | GACATGAGAGTCCTCGCTCAG | hs_Vk1_13_lead_in | TCAGCTCCTGGGGCTTCT |
| hs VK3 leader | ATGGAAGCCCCAGCTCAG | hs_Vk3_lead_in | TCTCTTCCTCCTGCTACTCTGG |
| hs VK3_15 leader | CCAGCGCAGCTTCTCTTC | hs_Vk4_1_lead_in | TCTGTTGCTCTGGATCTCTGG |
| hs VK4_1 leader | ATGGTGTTGCAGACCCAGGT | hs_Vk5-2_lead_in | TCCTCCTCCTTTGGATCTCTG |
| hs VK5_2 leader | TCCCAGGTTCACCTCCTCAG |  |  |
| ***Reverse primers*** | | ***Reverse primers*** | |
| Cκ out | GTTTCTCGTAGTCTGCTTTGCTCA | Cκ inner | CCTGGGAGTTACCCGATTGG |
