# Supplementary material for: Antigen-specific single B cell sorting and expression-cloning from immunoglobulin humanized rats: a rapid and versatile method for the generation of high affinity and discriminative human monoclonal antibodies
Source: BMC Biotechnol. 2017 Jan 9;17:3. doi: 10.1186/s12896-016-0322-5 (PMC5234254; doi:10.1186/s12896-016-0322-5)
Supplement: Additional file 2: Table S1. — Results of Ag-specific Ig genes sequencing. (DOCX 113 kb) [file 12896_2016_322_MOESM2_ESM.docx]

**Supplemental Table 1 – Results of Ag-specific Ig genes sequencing**

|  |  | PCR | | Heavy chain | | | | | | | Light chain | | | | Specificity | Affinity |
| --- | --- | --- | --- | --- | --- | --- | --- | --- | --- | --- | --- | --- | --- | --- | --- | --- |
| antigen | Name | gamma | VL | V-gene | V mutations aa | J-gene | D-gene | VH CDR | AA junction | gamma | V-gene | V mutations aa | J-gene | AA junction |  |  |
| β-Galactosidase | 2_C1 | + | + | IGHV3-23 | 5 | IGHJ4 | IGHD3-16 | [8.8.17] | CAKGVMITFGGVIVPFDYW | 1 | IGLV3-1 | 0 | IGLJ2*01 | CQAWDSNTAVF | yes | Not detectable |
|  | 2_D1 | + | + | IGHV4-39 | 3 | IGHJ5 | IGHD1-26 | [10.7.17] | CARHGGLYSGSYSNWFDPW | 1 | IGLV3-1 | 0 | IGLJ2*01 | CQAWDSSTVVF | yes | 0.56.10^-9^ |
|  | 2_E1 | + | + | IGHV3-23 | 1 | IGHJ6 | IGHD1-26 | [8.8.16] | CAKDRVVGATTGYDMDVW | 1 | IGLV3-1 | 3 | IGLJ2*01 | CQAWDSNTVVF | yes | 3.62.10^-7^ |
|  | 2_G1 | + | + | IGHV4-39 | 3 | IGHJ3 | IGHD1-26 | [10.7.15] | CARQSIVGATLDAFDIW | 1 | IGLV3-19 | 0 | IGLJ2*01 | CNSRDSSGNRVVF | yes | 1.44.10^-9^ |
|  | 2_H1 | + | + | IGHV3-23 | 8 | IGHJ4 | IGHD3-10 | [8.8.15] | CAKDQYYGSGSQAFDYW | 1 |  |  |  |  | yes | Not detectable |
|  | 2_E2 | + | + | IGHV3-7 | 2 | IGHJ4 | IGHD3-9 | [8.8.15] | CATSIYDILTGYLFDYW | 2b | IGLV3-19 | 4 | IGLJ2*01 | CNSRDSSGNHVVF | yes | Not detectable |
|  | 2_F2 | + | + | IGHV3-23 | 2 | IGHJ6 | IGHD1-1 | [8.8.14] | CAKVQTGTTGYGMDVW | 1 |  |  |  |  | yes | 1.82.10^-9^ |
|  | 2_G2 | + | + | IGHV3-33 | 1 | IGHJ4 | IGHD4-17 | [8.8.9] | CARTTGKFDYW | 1 | IGLV3-21 | 0 | IGLJ2 or IGLJ3 | CQVWDSSSDHSVF | yes | Not detectable |
| CD22-Fc | 4D1 | + | + | IGHV4-31 | 2 | IGHJ4 | IGHD1-26 | [10.7.11] | CAREVGPIIVGYW | 1 or 2a | IGKV1-12 | 0 | IGLJ4 | CQQANSFPLTF | No | Not determined |
|  | 4E1 | + | + | IGHV4-39 | 7 | IGHJ2 | IGHD1-26 | [10.7.17] | CARHFVGPTIPDYWYFDLW | 1 or 2a | IGKV1-16 | 0 | IGLJ4 | CQQYNSYPLTF | No | Not determined |
|  | 4F2 | + | + | IGHV3-9 | 2 | IGHJ4 | IGHD2-8 | [5.8.13] | CARERDWGWYYFDYW | 1 or 2a | IGKV4-1 | 3 | IGKV4 | CQQYYSTPLTF | No | Not determined |
|  | 5E2 | + | + | IGHV3-9 | 3 | IGHJ6 | IGHD7-27 | [8.8.14] | CAKDLGMNYYFGLDVW | 1 or 2a | IGKV4-1 | 3 | IGKV4-1 | CQQYYSTPFTF | Yes  Fc specific | Not determined |
|  | 4C1 | + | + | IGHV3-9 | 6 | IGHJ6 | IGHD7-27 | [8.8.14] | CAKDLGMNYYFGMDVW | 1 or 2a | IGKV4-1 | 3 | IGKV4-1 | CQQYYSTPFTF | Yes Fc specific | Not determined |
|  | 5E1 | + | + | IGHV6-1 | 1 | IGHJ4 | IGHD1-26 | [10.9.14] | CARDTYSGSYLPFDYW | 1 or 2a | IGKV4-1 | 3 | IGKV4-1 | CQQYYSTPYTF | Yes CD22 specific | 16.17.10^-9^ |
| HLA-A | 1.2 | + | + | IGHV4-39 | 3 | IGHJ4 | IGHD6-19 | [10.7.14] | CARGHSSGWTYYFDYW | 1 or 2a | IGKV1-5 | 2 | IGKJ4 | CQQYYSYTF | Yes | Not determined  (weak production) |
|  | 1.3 | + | + | IGHV4-39 | 3 | IGHJ4 | IGHD6-19 | [10.7.14] | CTRGHSSGWTYYFDYW | 1 or 2a | IGKV1-5 | 2 | IGKJ4 | CQQYYSYTF | No | Not determined |
|  | 1.4 | + | + | IGHV4-39 | 1 | IGHJ4 | IGHD3-22 | [10.7.14] | CARHYYYDSSGSFDYW | 1 or 2a | IGKV1-17 | 1 | IGKJ1 | CLQHNSYPWTF | Yes | Not determined |
|  | 1.5 | + | + | IGHV4-39 | 3 | IGHJ4 | IGHD6-19 | [10.7.14] | CTRGHSSGWTYYFDYW | 1 or 2a | IGKV1-5 | 2 | IGKJ4 | CQQYYSYTF | Yes | 1.27.10^-8^ |
|  | 1.6 | + | + | IGHV3-23 | 0 | IGHJ6 | IGHD3-10 | [8.8.16] | CAKGDTMVRGVPYGMDVW | 1 or 2a | IGKV1-12 | 1 | IGKJ5 | CQQANSFPITF | Yes | Not determined  (weak production) |
|  | 1.7 | + | + | IGHV4-39 | 0 | IGHJ4 | IGH6-19 | [10.7.14] | CARGHSSGWTYYFDYW | 1 or 2a | IGKV1-5 | 3 | IGKJ4 | CQQYYSYTF | Yes | Not determined  (Unstable Ab) |
| HLA-A2/pp65 | 1.2 | + | + | IGHV4-34 | 3 | IGHJ4 | IGHD2-2 | [8.7.11] | CARGYNMVGFDYW | 1 or 2a | IGLV3-1 | 0 | IGLJ1 | CQAWDSSTYVF | Yes | Not detectable |
|  | 1.3 | + | + | IGHV3-23 | 4 | IGHJ5 | IGHD6-13 | [8.8.12] | CATY(H)STSGGWFDPW | 1 or 2a | IGKV1-12 | 0 | IGKJ4 | CQQANSFPLTF | No | Not determined |
|  | 1.4 | + | + | IGHV3-30 | 0 | IGHJ5 | IGHD1-26 | [x.8.14] | CARSYSGSYTNWFDPW | 1 or 2a | IGKV1-12 | 3 | IGKJ4 | CQQANSFPLTF | No | Not determined |
|  | 1.5 | + | + | IGHV4-34 | 1 | IGHJ4 | IGH6-13 | [8.7.11] | CARGYSMVGFDYW | 1 or 2a | IGLV3-1 | 0 | IGLJ1 | CQAWDSSTYVF | Yes | 5.94.10^-8^ |
|  | 2.2 | + | + | IGHV3-23 | 2 | IGHJ3 | IGHD1-20 | [8.8.14] | CAKEGITGTTGAFDIW | 1 or 2a | IGLV3-10 | 0 | IGLJ3 | CYSTDSSGNRVF | No | Not determined |
|  | 2.3 | + | + | IGHV3-33 | 1 | IGHJ4 | IGH3-10 | [8.8.14] | CAREHSYGSGNYVDYW | 1 or 2a | IGLV3-10 | 1 | IGLJ3 | CYSTDSSGNHRVF | No | Not determined |
|  | 2.4 | + | + | IGHV3-23 | 4 | IGHJ4 | IGHD6-19 | [8.8.13] | CATPDRSSGWSFDYW | 1 or 2a | IGLV2-13 or 2-5 | 6 | IGLJ2 | CCSYAGSSTFVVF | No | Not determined |
|  | 2.7 | + | + | IGHV4-34 | 0 | IGHJ4 | IGHD5-18 | [8.7.11] | CARGYSMVGFDYW | 1 or 2a | IGLV3-1 | 0 | IGLJ1 | CQAWDSSTYVF | Yes | 1.44.10^-7^ |
|  | 3.6 | + | + | IGHV3-23 | 3 | IGHJ4 | IGHD6-19 | [8.8.13] | CATPDRSSGWSFDYW | 1 or 2a | IGLV2-23 | 1 | IGLJ2 | CCSYAGSSTFVVF | No | Not determined |
