# Supplementary material for: Antigen-specific single B cell sorting and expression-cloning from immunoglobulin humanized rats: a rapid and versatile method for the generation of high affinity and discriminative human monoclonal antibodies
Source: BMC Biotechnol. 2017 Jan 9;17:3. doi: 10.1186/s12896-016-0322-5 (PMC5234254; doi:10.1186/s12896-016-0322-5)
Supplement: Additional file 3: Table S2. — Results of CD22-specific Ig genes sequencing. (DOCX 141 kb) [file 12896_2016_322_MOESM3_ESM.docx]

**Supplemental Table 2 – Results of CD22-specific Ig genes sequencing**

|  |  | Heavy chain | | | | | Light chain | | | | Specificity |
| --- | --- | --- | --- | --- | --- | --- | --- | --- | --- | --- | --- |
| Name | | V-gene | D-gene | J-gene | V mutations aa | AA junction | V-gene | J-gene | V mutations aa | AA junction |  |
| γ1λ1 | | IGHV3-11 | D6-19 | J5 | D36N, Y67N | CARDESSSGLW | IGLV3-1 | J2 or J3 | D36E | CQAWDRRTAVF | Yes |
| γ2λ2 | | IGHV4-39 | D1-26 | J2 | S64T, T77I, K90R | CVRQEVGPTGWFWYFDLW | IGLV3-10 | J2 or J3 |  | CYSTDSSGKVF | Yes |
| γ3λ3-5 | | IGHV4-39 | D6-13 | J2 |  | CASQYSSTWFWYFDLW | IGLV3-1 | J2 or J3 | V11A | CQAWDSSTAVF | Yes |
| γ4λ4-1 | | IGHV4-39 | D1-26 | J6 | S31N, S34N, S35G | CARCIVGATTIGMDVW | IGLV3-10 | J2 or J3 |  | CYSTDSSGNHRVF | Yes |
| γ4λ4-2 | | IGHV4-39 | D1-26 | J6 | S31N, S34N, S35G | CARCIVGATTIGMDVW | IGLV3-10 | J2 or J3 |  | CYSTDSSGNHRVF | No |
| γ7λ7 | | IGHV3-11 | D6-6 | J4 | I42T, S58F | CVRQEFVFDYW | IGLV3-1 | J2 or J3 | A39T, I91V | CQAWDSFTVVF | Yes |
| γ8λ8 | | IGHV3-11 | D3-10 | J4 | S26Y, V53I, S59G, Y66H, K84E, A96V | CARQEFMFDYW | IGLV3-1 | J2 or J3 | E74A, Q95H | CQAWDRLTVVF | Yes |
| γ11λ11 | | IGHV3-11 | D1-26 | J4 | G62S, Y66N | CARSDFLFDYW | IGLV3-10 | J2 or J3 | Y42N | CYSTDSSGNHVVF | No |
| γ12λ12 | | IGHV1-8 | D5-12 | J4 | A25T, Y28N, T82A, S85T, A87V | CARGWLRFDYW | IGLV2-23 | J2 or J3 | V30I, S35T, S65T, S77A, Y103H | CCSYAGRSTYVVS | No |
| γ13λ2 | | IGHV4-39 | D1-26 | J2 | T77I, K90R | CVRQEVGPTGWFWYFDLW | IGLV3-10 | J2 or J3 |  | CYSTDSSGKVF | No |
| γ14λ14-1 | | IGHV3-11 | D6-13 | J2 | A24S, T64I | CARLNSSSWDWYFDLW | IGLV3-10 | J3 |  | CYSTDSSGNHRVF | Yes |
| γ15λ15 | | IGHV4-39 | D3-10 | J4 | S34R, S55N | CAQVLWFGELLWDYW | IGLV3-19 | J2 or J3 | T22K, L52F | CNSRDSSGNHVVF | Yes |
| γ17λ17 | | IGHV3-11 | D2-21 | J3 | L19Q, Y38C, S58T, S59G, G62S, S63R, E97D | CARELDGFDIW | IGLV2-23 | J3 | S27N, N37H, Y42F, G57D, S65I, G84D, A96P, A100S | CCSYAGSATWVF | Yes |
| γ18λ18-3 | | IGHV3-9 | D1-1 | J6 | A24T, H40Y | CAKAIRYNWNDAYHYGLDVW | IGLV3-1 | J2 or J3 | K27E, S65T | CQAWDNSTVVF | Yes |
| γ19λ19-2 | | IGHV4-31 | D5-12 | J4 | Y38S, Y58S, S64I, T77N | CARTGDIVATYYFDYW | IGLV3-19 | J2 or J3 | I21V, I54F, N66S | CYSRDSSGDHVVF | Yes |
| γ20λ2 | | IGHV4-39 | D1-26 | J2 | S59G, S64I | CARHEVGATGWFWYFDLW | IGLV3-10 | J2 or J3 |  | CYSTDSSGKVF | No |
| γ21λ21 | | IGHV3-11 | D2-21 | J3 | S58P,G62S, S63I, Y66S, E97D | CARELDGFDIW | IGLV3-1 | J1 | Y38F | CQAWDTSTNYVF | Yes |
| γ22λ22 | | IGHV3-11 | D6-6 | J4 | S58F | CARQEFVFDYW | IGLV3-1 | J2 or J3 | S20T, K27T, A39T, I91V | CQAWDSFTVVF | Yes |
| γ24λ23 | | IGHV3-9 | D5-18 | J4 | G63H, T99A, L101F | CAKASGYSYVDW | IGLV2-14 | J2 or J3 | N37S, E56D | CSSYTSSGTLVF | Yes |
| γ25λ24 | | IGHV3-33 | D1-26 | J6 | G38V, K65E, S93R | CARDPPWELLNNYYGMDVW | IGLV3-10 | J2 or J3 | A100G | CYSTDSSGVVF | Yes |
| γ26λ25 | | IGHV3-9 | D1-26 | J4 |  | CAKDGGTYSGSFDYW | IGLV2-23 | J3 | S27N, N37H, Y42F, G57D, S65I, V71I, G84D, A96P, A100S | CCSYAGSATWVF | Yes |
| γ27λ26 | | IGHV3-11 | D1-26 | J4 | S58T, S59G, G62S, Y66N | CARSDFLFDYW | IGLV3-1 | J2 or J3 | Y38F | CQAWDSISVVF | Yes |
| γ28λ27 | | IGHV3-23 | D3-22 | J2 | A55T, G63V | CAKELDDSSGYPYWYFDLW | IGLV3-1 | J2 or J3 | D26N | CQAWDSSTVVF | Yes |
| γ31λ30-1 | | IGHV3-30 | D1-26 | J4 | N64V, N85D | CAKPSGSYSFDYW | IGLV3-1 | J1 | K37T, Y42F | CQAWDSSTYVF | No |
| γ33λ32-6 | | IGHV4-31 | D6-13 | J3 | S16A, Q17E, T24I, S34R, G36A, S40N, I42L, Y58H, V76L, V80I, K90R | CARDIAVLGPDAFDIW | IGLV3-1 | J2 or J3 | R75Q | CQAWDNRVF | No |
| γ34λ33 | | IGHV3-23 | D3-22 | J2 | E6D, A38T, G63S | CAKELDDSSGYPYWYFDLW | IGLV3-1 | J2 or J3 | T88S | CQAWDSSTVVF | Yes |
| γ35λ34 | | IGHV4-39 | D3-3 | J2 | L4V, G28A, S31R, S36G, Y37F, Y38F, Y67N | CARLSPGFVWYFDLW | IGLV3-19 | J2 or J3 | Y55C | CNSRDSSGNHVVF | Yes |
| γ38λ37 | | IGHV1-8 | D6-19 | J4 | Q1E, I39L | CARERVTVAGPFDYW | IGLV2-14 | J2 or J3 | G35A, E56D | CSSYTSSNTLIF | No |
| γ40λ2 | | IGHV4-39 | D1-26 | J2 | Y37C, S59G, Y67F, A100T | CARHEVGATGWFWYFDLW | IGLV3-10 | J2 or J3 |  | CYSTDSSGKVF | No |
| γ41λ40 | | IGHV4-39 |  | J1 | K14R, I53L, G54A, S55T, S59N, Y66F, Y67C, S79P, S92T | CAREYFHHW | IGLV2-23 | J3 | N37H, Y42F, Q44H, G57D, S65I, G84D, A100S | CCSYAGSGTWVF | Yes |
| γ42λ41 | | IGHV3-11 | D6-6 | J4 | S58F, A96G | CARQEFVFDYW | IGLV3-1 | J2 or J3 | A39T, V51L, I91V | CQAWDSFTVVF | Yes |
| γ43-2λ17 | | IGHV3-33 | D3-22 | J5 | S36G, G38V, W41G, N64D, K84R | CARDRSGNYLNWFDPW | IGLV2-23 | J3 | S27N, N37H, Y42F, G57D, S65I, G84D, A96P, A100S | CCSYAGSATWVF | No |
| γ43-4λ17 | | IGHV4-39 |  | J1 | K14R, I53L, G54A, S55T, S59N, Y66F, Y67C, V76L, S79P, S92T | CAREYFHHW | IGLV2-23 | J3 | S27N, N37H, Y42F, G57D, S65I, G84D, A96P, A100S | CCSYAGSATWVF | Yes |
| γ44λ17 | | IGHV4-31 | D5-12 | J4 | S64N, Y67H, V76A | CARVGDVVATFFFDYW | IGLV2-23 | J3 | S27N, N37H, Y42F, G57D, S65I, G84D, A96P, A100S | CCSYAGSATWVF | No |
| γ45λ44-3 | | IGHV3-23 | D6-13 | J4 | S36T, A55G | CAKSPWYFDYW | IGLV2-23 | J2 or J3 | T26I, L52V | CCSYAGSSTFVF | Yes |
| γ46λ45-2 | | IGHV4-39 | D1-26 | J2 |  | CARHEVGATGWFWYFDLW | IGLV3-10 | J2 or J3 | Y38D, I91V | CYSTDSSGKVF |  |
| γ47λ46 | | IGHV4-34 | D3-9 | J6 | P15S, S40N, P45S, S64T, K72R, T99S | CARDNDILTGPVYYYGMAVW | IGLV3-19 | J2 or J3 | I21L, S36V, Y37F, S40A, P46A, N66I, S79T, S80A, A94T, D101E, Y103H | CNSRDKSADLLLF | No |
| γ23κ5-2 | | IGHV3-9 | D5-18 | J4 | G27E, N59S | CARDRGYSYGAFDYW | IGKV1-5 | J2 | S69R | CQQYNSHSCSF | Yes |
| γ26κ6 | | IGHV3-9 | D1-26 | J4 |  | CAKDGGTYSGSFDYW | IGKV1-5 | J2 | S36N, S37N | CQQYNSYSCSF | Yes |
| γ29κ7 | | IGHV4-34 | D3-9 | J4 | S35D, G36S, S64N, T77S, V80I | CAGTFYDILTGYYPLGYW | IGKV1-9 | J1 | Y103H | CQQLNSYPRTF | No |
| γ30-1κ7 | | IGHV3-30 | D1-26 | J4 | N64V, N85D | CAKPSGSYSFDYW | IGKV1-9 | J1 | Y103H | CQQLNSYPRTF | No |
| γ30-2κ7 | | IGHV4-4 | D3-10 | J4 |  | CARDDYFYGSGSYYNYW | IGKV1-9 | J1 | Y103H | CQQLNSYPRTF | Yes |
| γ32κ7 | | IGHV4-31 | D6-13 | J3 | S16A, Q17E, T24I, S34R, G36A, S40N, I42L, Y58H, V76L, V80I, K90R | CARDIAVLGPDAFDIW | IGKV3-15 | J4 | S7T, A19V, T90A, Y103F | CQHYKNWPLTF | No |
| γ39κ10 | | IGHV4-31 | D3-10 | J4 | S36I, Y55N, Y57F, S59T, N68I, S92N | CARAGSGRPFDYW | IGKV1-5 | J2 | S14N | CQQYNSLCSF | No |
