# Supplementary material for: Antigen-specific single B cell sorting and expression-cloning from immunoglobulin humanized rats: a rapid and versatile method for the generation of high affinity and discriminative human monoclonal antibodies
Source: BMC Biotechnol. 2017 Jan 9;17:3. doi: 10.1186/s12896-016-0322-5 (PMC5234254; doi:10.1186/s12896-016-0322-5)
Supplement: Additional file 4: — Analysis of anti-human CD22 human mAbs affinity and specificity on human PBMCs. a) Affinity determination of anti-human CD22 mAbs using SPR by flowing various concentration of CD22 antibody over CD22 chip-bound. b) Flow cytometry analysis of CD22 mAbs on human PBMC. Human PBMC were labeled with anti human CD19 (APC) and purified CD22 mAbs (clone γ1λ1, γ3λ3-5, γ23κ5-2 or γ27λ26) coupled with Alexa Fluor 568 fluorochrome or with a commercial mouse mAb anti-human CD22 (Ms anti-human CD22). (PPT 666 kb) [file 12896_2016_322_MOESM4_ESM.ppt]

## Slide 1
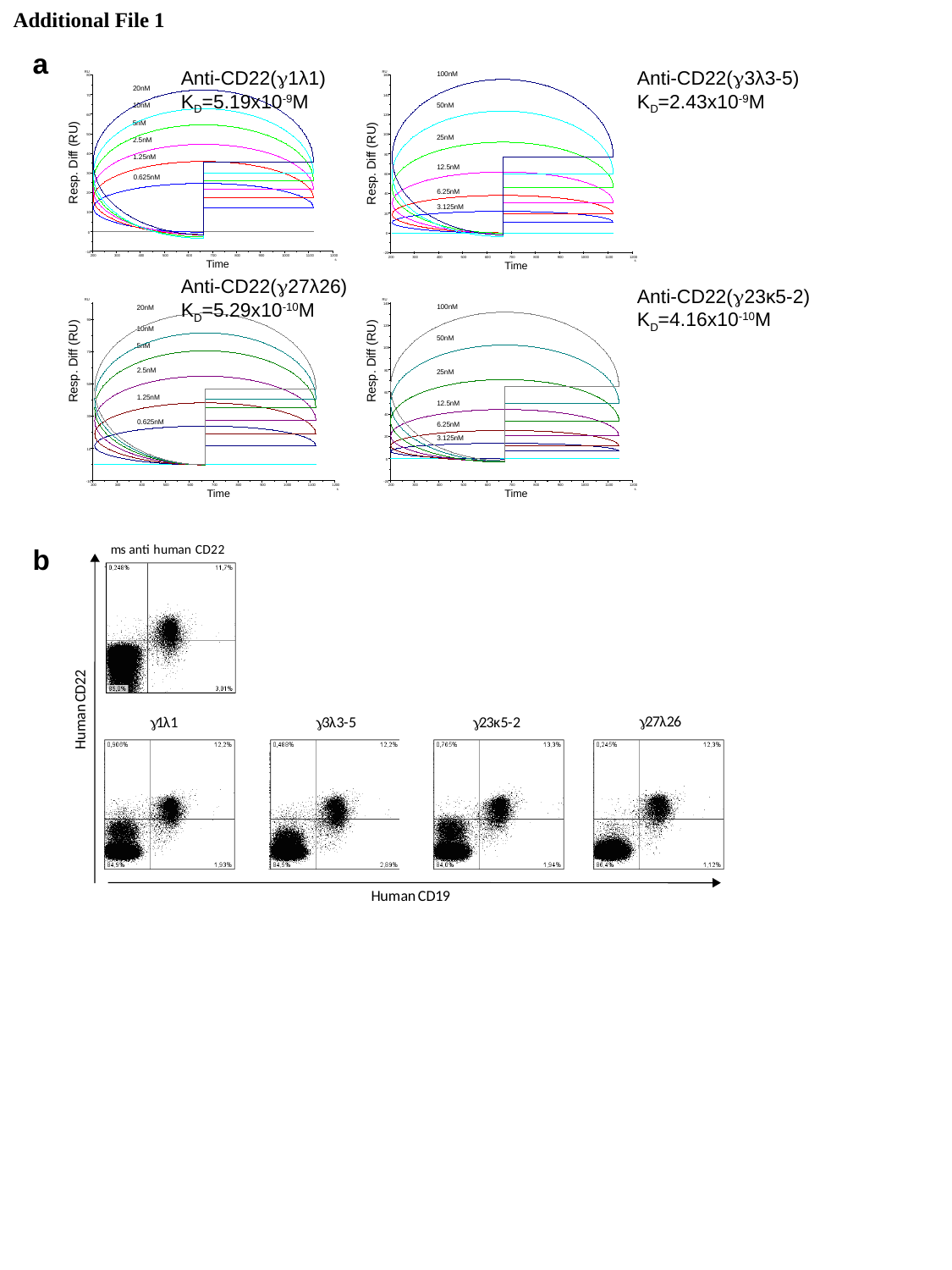

Additional File 1
a
Anti-CD22(γ1λ1)
KD=5.19x10^-9^M
Anti-CD22(γ3λ3-5)
KD=2.43x10^-9^M
RU
80
20nM
70
10nM
60
5nM
50
2.5nM
40
1.25nM
30
0.625nM
20
10
0
-10
200
300
400
500
600
700
800
900
1000
1100
1200
Time
s
RU
100nM
160
140
50nM
120
100
25nM
80
12.5nM
60
6.25nM
40
3.125nM
20
0
-20
200
300
400
500
600
700
800
900
1000
1100
1200
Time
s
Resp. Diff (RU)
Resp. Diff (RU)
Anti-CD22(γ27λ26)
KD=5.29x10^-10^M
Anti-CD22(γ23κ5-2)
KD=4.16x10^-10^M
Resp. Diff (RU)
Resp. Diff (RU)
RU
20nM
90
10nM
5nM
70
2.5nM
50
1.25nM
30
0.625nM
10
-10
200
300
400
500
600
700
800
900
1000
1100
1200
Time
s
RU
140
100nM
120
50nM
100
25nM
80
60
12.5nM
40
6.25nM
3.125nM
20
0
-20
200
300
400
500
600
700
800
900
1000
1100
1200
Time
s
b
